# Phytosynthesis of nanoparticles: concept, controversy and application

**DOI:** 10.1186/1556-276X-9-229

**Published:** 2014-05-12

**Authors:** Azamal Husen, Khwaja Salahuddin Siddiqi

**Affiliations:** 1 Department of Biology, College of Natural and Computational Sciences, University of Gondar, P.O. Box 196, Gondar, Ethiopia; 2 Department of Chemistry, College of Natural and Computational Sciences, University of Gondar, P.O. Box 196, Gondar, Ethiopia

**Keywords:** Metal, Metal oxide, Carbon nanomaterials, Green synthesis, Agriculture, Medicine

## Abstract

Nanotechnology is an exciting and powerful discipline of science; the altered properties of which have offered many new and profitable products and applications. Agriculture, food and medicine sector industries have been investing more in nanotechnology research. Plants or their extracts provide a biological synthesis route of several metallic nanoparticles which is more eco-friendly and allows a controlled synthesis with well-defined size and shape. The rapid drug delivery in the presence of a carrier is a recent development to treat patients with nanoparticles of certain metals. The engineered nanoparticles are more useful in increasing the crop production, although this issue is still in infancy. This is simply due to the unprecedented and unforeseen health hazard and environmental concern. The well-known metal ions such as zinc, iron and copper are essential constituents of several enzymes found in the human system even though the indiscriminate use of similar other metal nanoparticle in food and medicine without clinical trial is not advisable. This review is intended to describe the novel phytosynthesis of metal and metal oxide nanoparticles with regard to their shape, size, structure and diverse application in almost all fields of medicine, agriculture and technology. We have also emphasized the concept and controversial mechanism of green synthesis of nanoparticles.

## Review

### Introduction

Globally, incredible changes in agricultural production patterns have taken place. It has become possible only through the application of modern labour saving technologies for intensive on-farm mechanization, irrigation, postharvest handling and use of improved crop varieties. Despite the tremendous progress made in agricultural productivity, still there exists food insecurity and poverty in many developing countries. Nanotechnology is an area which includes almost all branches of science including biology, chemistry, physics, engineering and medicine, although the main thrust of research focuses on the food and agriculture. The products based on nanotechnologies were estimated to be more than 800 and expected to raise more in the market within the next few years [1,2]. By next year, it is expected that more than 15% of all products on the global market will have some kind of nanotechnology incorporated into their manufacturing process [3].

The major global problem is to increase food production with limited resources and minimum and efficient use of fertilizer and pesticides without polluting the environment. A variety of nanomaterials have been tested against germination of seeds, growth of shoot/root and crop production besides testing their adverse effect on the flora and fauna. The Food and Agriculture Organization (FAO) of the United Nations and World Health Organization (WHO) at their expert meeting on the ‘application of nanotechnologies in the food and agriculture sectors’ in Rome in 2010 have identified the potential of nanotechnology in food and agriculture sectors and are investing heavily in its application to food production at a global level [4]. It was aimed at developing innovative ways to increase food production, water treatment, preservation and packaging besides toxicology and human health risk associated with the use of nanotechnology. Since the engineered nanoparticles of 1- to 100 nm may have different physical and chemical properties than the naturally occurring ones, their impact on human health must be assessed as a function of their size and shape. The committee recognized the potential risk and benefits of nanotechnology but wanted the sponsored researchers to address these issues in their ongoing projects. The global market in nanotechnology is expected to reach US$1 trillion by 2015 [5].

Plants are able to hyperaccumulate metals, up to concentrations several hundreds of times those found in non-hyperaccumulating plants [6-8]. It is thought that this provides a measure of protection for the plant from insects and other herbivores. The use of nanoparticles in the growth of plants and control of plant diseases is a recent practice [9-13]. Nanomaterial can be used in the diagnosis of some plant diseases by labelled nanoparticles. It can be helpful in the increased production of useful small edible plants such as spinach, radish, rye or grain like maize, rice and wheat [14]. Nanotechnology has potential for the controlled release of drug, nutrients and pesticides/agrochemicals for efficient use of trace elements without disturbing the non-target insects [15]. It also provides way to convert organic wastes to useful products [15,16]. Porous hollow silica nanoparticles are used for the controlled delivery of the water-soluble pesticide validamycin [17]. Biodegradable organic waste and plant or fruit peeling have also been used for the synthesis of nanoparticles as all of these contain phenols, flavonoids and reducing agents [18-20]. Nanoparticles reveal completely new or improved properties based on specific characteristics such as size, distribution and morphology, if compared with larger particles of the bulk material they are made of [21]. Since the absorption of minerals by the plant is non-selective, some of the metal ions in conjunction with anions may cause toxicity if they exceed the tolerance limit of the plant. When the nanoparticles are absorbed, they are subsequently translocated and accumulated in different parts of the plants forming complex with carrier proteins. It is, however, not yet clear as to how some plant species select certain nanoparticles and reject others. If they are larger than the pore of root, they get accumulated at the surface, and when they are smaller, they get absorbed and transported to other parts of the plants.

**Figure 1:**
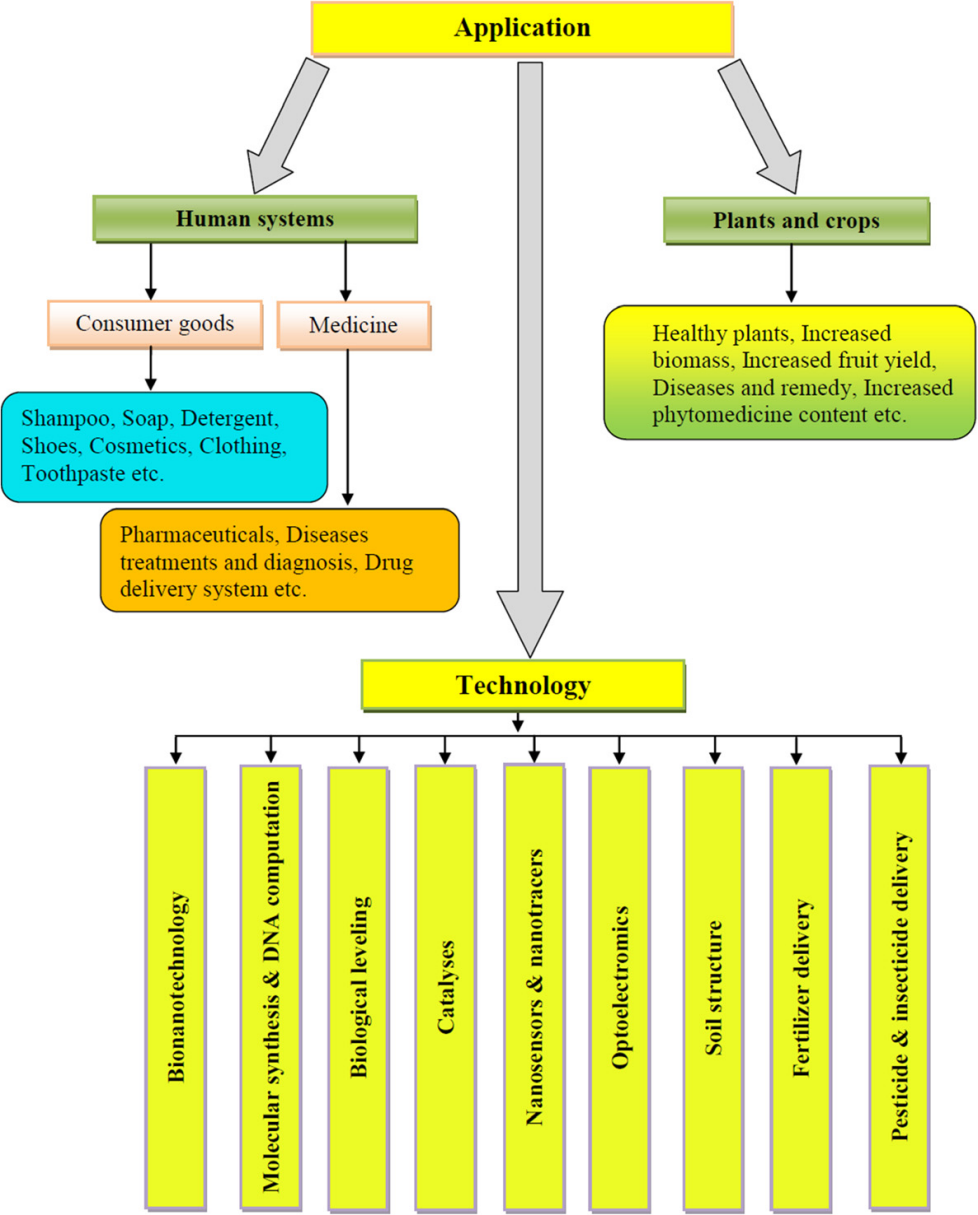
Application of engineered nanoparticles in living systems.

**Figure 2:**
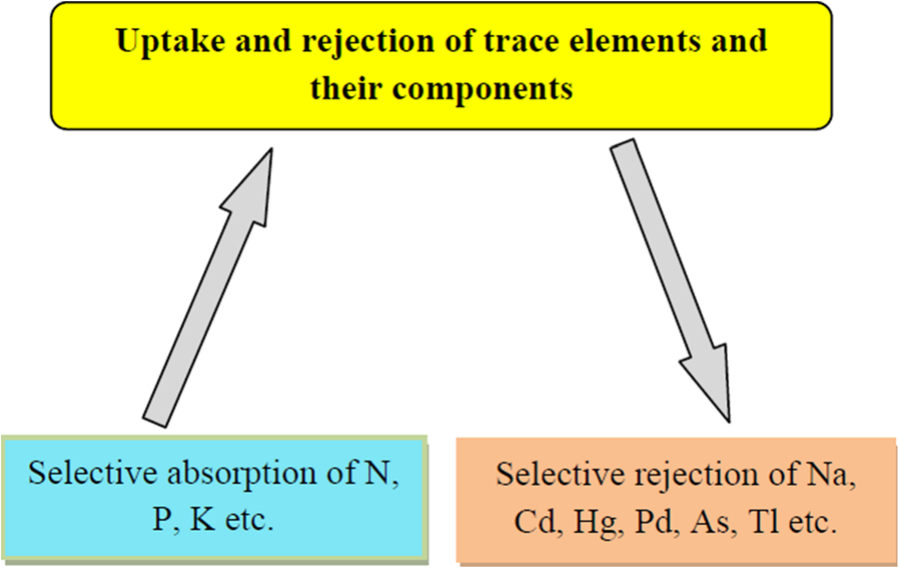
Selective absorption and rejection of nanoparticles.

It is the present requirement to produce more food crops from the extant resources. Genetically modified crops are a way to substantially produce better food grain, but it has some implications [22]. The production of food crop from engineered nanoparticle is another alternative. A wide range of metal oxide nanoparticles (ZnO, TiO_2_, Al_2_O_3_, FeO, Fe_2_O_3_, etc.), fullerenes, carbon nanotubes, quantum dots, etc. have an increasing range of applications (Figure 1) for different purposes [23] and make their way easily in the environment [24,25]. Their potential adverse effects on the environment and human health are being subjected to intense debate [26]. Although nanoparticles, whether natural or synthetic, are being used in every sphere of life, their exploitation in agriculture is limited. Studies have been directed towards seed germination, root elongation, foliar growth and seed and crop development [27]. The use of nanoparticles without knowing the toxic effect on the plant may sometimes cause mutation, which may be very damaging to both plants and ecosystem. Nanoparticles when sprayed or inoculated will penetrate and transported to various parts of the plant. Some nanoparticles are stored in extracellular space and some within the cell. Some plants reject the nanoparticles and some accept or store them (Figure 2). Inadvertent use of rare and precious metal nanoparticles generally does not show any positive effect on the plant except for their storage and blocking the passage of vessels [28-30]. The process of nanoparticle accumulation in plants may be used to clean up nanoparticle contamination and extraction of metal from such plants. The extraction of metal from such plants is called phytomining or phytoextraction [6,31,32]. An et al. [33] have reported an increase in ascorbate and chlorophyll contents in leaves of asparagus treated with silver nanoparticles. Likewise, soybean treated with nano-iron showed increased weight of beans [34]. The result is not always positive as in some cases no effect or negative effect was noted when the plants were treated with gold, silver or copper nanoparticles [35,36].

Nanoparticles of commercial importance are being synthesized directly from metal or metal salts, in the presence of some organic material or plant extract. The creepers and many other plants exude an organic material, probably a polysaccharide with some resin, which help plants to climb vertically or through adventitious roots to produce nanoparticles of the trace elements present, so that they may be absorbed. One such example comes from English ivy (*Hedera helix*) which produces from its adventitious root hairs' nanocomposite adhesive that contains spherical nanoparticles of 60- to 85 nm diameter. The production of the nanoparticles depends on the proliferation of the adventitious roots. Usually, indole-3-butyric acid (IBA) and α-naphthalene acetic acid (NAA) have been recommended for promoting adventitious roots in shoot cutting propagation in many shrub [37-39] or tree [40-42]. In order to increase the proliferation of the root to produce larger quantity of the composite nanomaterial from English ivy, an auxin namely IBA was used as a root growth enhancer. Maximum root production was achieved by soaking the shoot segments of the climber in 0.1 mg mL^-1^ IBA [43]. It is worth mentioning that the adventitious root hairs which do not come in touch with the solid surface dry up and abort. The overall production of the composite nanomaterial is only 0.75% which is sufficient to support the plant. It is uncertain whether such material can be used for the production of metal nanoparticles as these are nanomaterial themselves. However, it may be used in hardening and cementing the teeth because it dries up quickly. Further studies from the plant resin and gums may enhance our knowledge in this area.

This review is intended to discuss the phytosynthesis of metal and metal oxide nanoparticles including carbon nanomaterials and their application in agriculture, medicine and technology.

### Engineered nanoparticles

**Figure 3:**
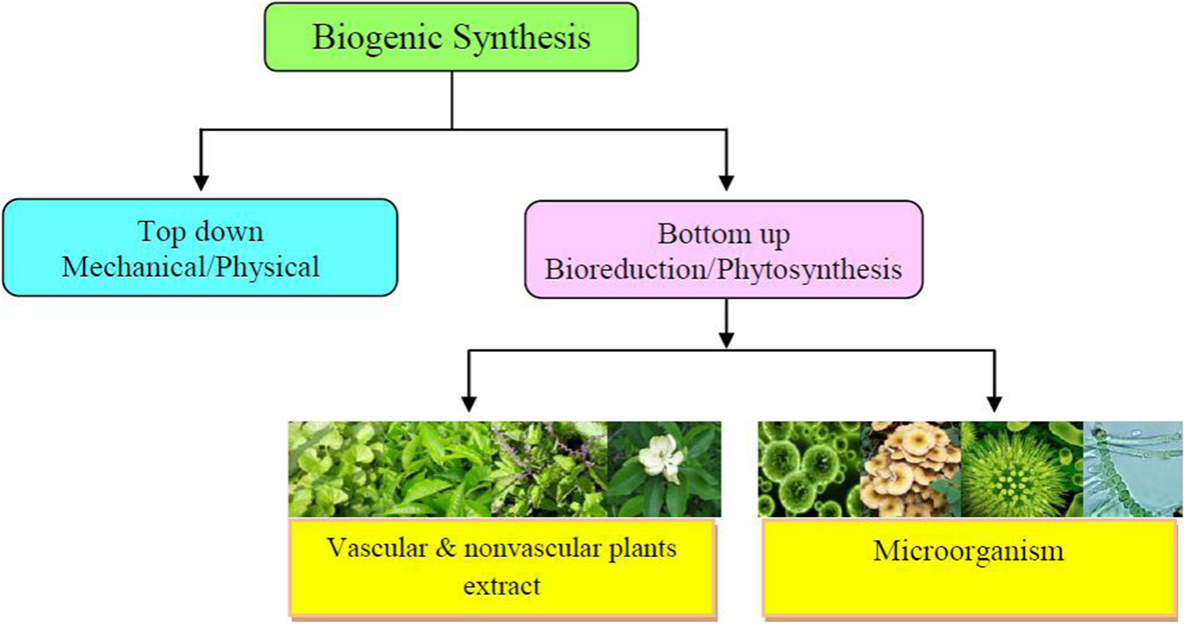
Flow diagram for biogenic synthesis of nanoparticles.

**Figure 4:**
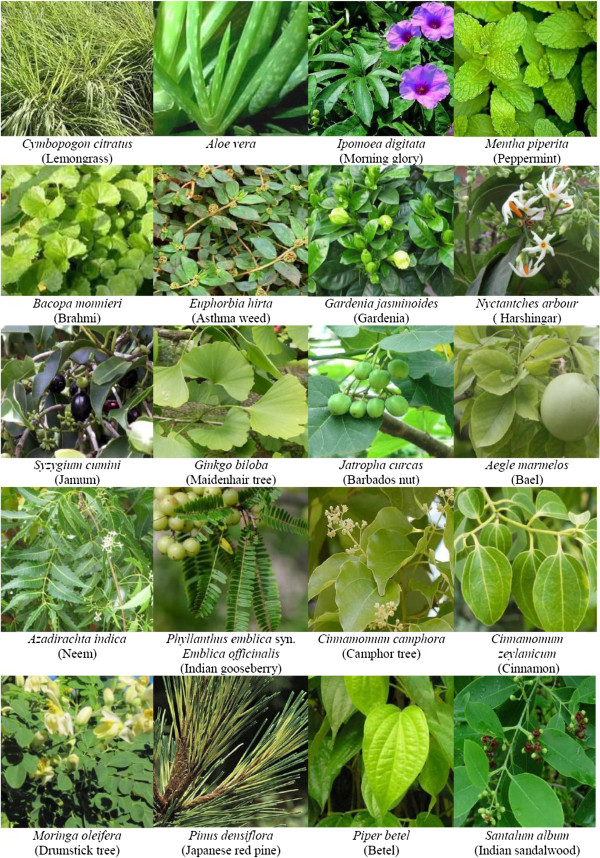
Herbs, shrubs and trees for nanoparticle fabrication.

The synthesis of nanoparticles (Figure 3) and their application in allied field has become the favourite pursuit of all scientists including biologist, chemists and engineers. It is known that almost all plants (herbs, shrubs or trees) containing aroma, latex, flavonoids, phenols, alcohols and proteins can produce metal nanoparticles from the metal salts (Figure 4). Although nanoparticles can be chemically synthesized by conventional methods, biosynthesis prevents the atmosphere from pollution. The shape and size of nanoparticles may be controlled and a desired type of nanoparticle may be produced by controlling the temperature and concentration of the medium. Engineered nanoparticles may be classified into the metal (or non-metal) and metal oxide nanoparticles.

### Metal nanoparticles

Synthesis of engineered nanoparticles is usually done by the interaction of microorganisms, algae or plant extracts. It is quite obvious that nanomaterials may be useful or harmful in living system depending on their shape, size and above all the nature of specific metal ion. The effect of engineered metal nanoparticles of varying size and concentration on different parts of a variety of plants is given in Table 1.

**Table 1:** Effects of engineered metal nanoparticles on plants

**Nanoparticle**	**Size (nm)**	**Plant**	**Concentration**	**Effect**	**References**
Aluminium		Corn, cucumber, lettuce, radish, rapeseed	2,000 mg L^-1^	No effect on germination	[44]
	1 to 100	Red kidney beans, ryegrass	10, 100, 1,000 and 10,000 mg L^-1^	No toxicity	[45]
		Radish, rapeseed	2,000 mg L^-1^	Improved root growth	[44]
		Ryegrass	2,000 mg L^-1^	Decreased root length	[44]
		Ryegrass	2,000 mg L^-1^	Reduced germination	[44]
		Corn, lettuce	2,000 mg L^-1^	Reduced root length	[44]
Copper		Lettuce	0.013% (*w*/*w*)	No effect on germination, improved shoot/root ratio	[13]
		Mung bean	<200 mg L^-1^	Reduced seedling growth	[30]
		Mung bean	800 mg L^-1^	Reduced shoot growth	[30]
		Wheat	<200 mg L^-1^	Reduced root and seedling growth	[30]
	50	Zucchini	1,000 mg L^-1^	Reduced biomass	[46]
	50	Zucchini	1,000 mg L^-1^	Reduced root growth	[46]
Dodecanethiol-functionalized gold		Lettuce	0.013% (*w*/*w*)	No effect on germination, improved shoot/root ratio	[13]
Gold	10	Cucumber, lettuce	62, 100 and 116 mg L^-1^	Positive effect on germination index	[47]
Iron		Flax, meadow fescue, red clover, white clover	100, 250 and 500 mg L^-1^	No effect on germination	[48]
		Barley, ryegrass	100 and 250 mg L^-1^	No effect on germination	[48]
		Barley, flax, ryegrass	2,000 and 5,000 mg L^-1^	Completely inhibited germination	[48]
		Barley	300 mg L^-1^	Reduced germination	[48]
		Flax, barley, ryegrass	>1,500 mg L^-1^	No germination	[48]
Mixture of gold/copper		Lettuce	0.013% (*w*/*w*)	No effect on germination, improved shoot/root ratio	[13]
Palladium entrapped in Al(OH)_2_ matrix		Lettuce	0.013% to 0.066% (*w*/*w*)	No effect on germination, improved shoot/root ratio	[13]
Silicon	10	Zucchini	1,000 mg L^-1^	Completely inhibited germination	[46]
Silver	20	Flax	20, 40, 60, 80 and 100 mg L^-1^	No effect on germination	[48]
	2	Cucumber, lettuce	62, 100 and 116 mg L^-1^	Low to zero toxicity	[47]
	20.6 ± 3.1	Clover	0.01 mg kg^-1^	Reduced aboveground biomass	[49]
			0.1 mg kg^-1^	No effect on biomass	[49]
			1 mg kg^-1^	No effect on biomass	[49]
	10	Wheat	0.5, 1.5, 2.5, 3.5 and 5.0 mg kg^-1^	Reduced shoot and root length	[50]
	5	Barley	10 mg L^-1^	Reduced germination	[48]
		Flax, barley	10 mg L^-1^	Reduced shoot length	[48]
	20	Barley	10 mg L^-1^	Reduced germination	[48]
		Barley	10 mg L^-1^	Reduced shoot length	[48]
		Barley, ryegrass	20 mg L^-1^	Reduced shoot length	[48]
	100	Zucchini	100, 500 and 1,000 mg L^-1^	Reduced transpiration	[46]
	100	Zucchini	500 and 1,000 mg L^-1^	Reduced biomass	[46]
	<100	Onion	100 mg L^-1^	Decreased mitosis, disturbed metaphase, sticky chromosome, cell wall disintegration and breaks	[51]
Silver colloidal form	0.6 to 2	Ryegrass	10 mg L^-1^	Reduced germination	[48]
		Ryegrass	20 mg L^-1^	Reduced germination	[48]
		Flax, ryegrass	10 mg L^-1^	Reduced shoot length	[48]
		Barley, flax, ryegrass	20 mg L^-1^	Reduced shoot length	[48]
Zinc		Corn, cucumber, lettuce, radish, rapeseed, ryegrass	2,000 mg L^-1^	Reduced root growth and elongation	[44]

The toxic metals like Cd, Hg, Pb and Tl will always produce toxic nanoparticles which may produce adverse effect in both plants and animals whether aquatic or terrestrial. However, several positive effects of engineered metal nanoparticles have been practically proved. Zn is known to be an essential element for both plants and animals. Since it is an essential constituent of over 30 enzymes, the activity of such metalloenzymes is lost during deficiency of the metal. It has always positive effect in the human system, provided it does not exceed the permissible limit. A suspension of 200 mg Zn L^-1^ showed phytotoxicity in certain vegetable plants [44], although such concentration is seldom attained in nature. It is clear that a concentration of up to 1 to 4 mg Zn L^-1^ does not exhibit any phytotoxicity which means that such results can be obtained only under experimental conditions. The phytotoxicity causes retardation in growth to the extent of plant being stunted. This effect can successfully be used in growing bonsai and ornamental plants on large scale. The effect that is produced after years of pruning the plants can be achieved in few months. Further, most frequently used engineered metal nanoparticles are discussed in the forthcoming sections.

#### ***Silver nanoparticles***

Silver nanoparticles may be used in cosmetics, food and medicine. The Ag nanocrystals or even the silver metal is known to possess antibacterial, antifungal and antioxidant properties [52-58]. They may also be useful in catalysis, although no specific reaction is known where Ag metal may have been used as a catalyst. The Ag nanoparticles or even silver nitrate is used in ointments to cure injury and burns as it prevents infection from spreading over the wound, increasing the surface area [59]. Unlike zinc oxide, silver has the inherent tendency to kill the bacteria without interacting deep into the cell wall of the microorganism. Zinc oxide, on the other hand, interacts with the enzyme present in the body cell which prevents further multiplication of microbes. Although the synthesis of nanoparticles using a variety of chemicals has become a focal theme in the recent time, biosynthesis of nanocrystals of varying shapes and sizes using plant extracts containing redox chemicals is prevalent. Such technologies need attention perhaps because they are environment friendly and prevent from further pollution by unwanted chemicals.

Antioxidant activity of a substance is defined as the removal of free radical before it causes oxidative damage to the living system. Ag nanoparticle is believed to be capped with the oxidized form of the functional groups present in the compounds in plant extract, thereby acting as antioxidant. It has been observed that the antioxidant action of capped Ag nanoparticles containing plant extract is higher than that of the plant extract alone [50,54]. Enhanced antimicrobial activity of Ag nanoparticles prepared from *Mimusops elengi* was reported against multi-drug resistant clinical isolates [60]. Ag nanoparticles synthesized from *Artemisia nilagirica *[61] and *Pongamia pinnata *[62] have also been found to be active against several microorganisms. Ag nanoparticles synthesized from *Morinda citrifolia* root extract have also exhibited cytotoxic effect on HeLa cell lines [63].

It is quite obvious that the plant extract certainly contains substantial quantity of benign chemicals which reduce the metal salt into nanocrystals. It has been practically determined that the quantity of *Cinnamomum camphora*, as reductant, is responsible for the size of nanocrystals of AgNO_3_. When 50 mL solution of 1 mM AgNO_3_ is exposed to as little as 0.1 g of biomass of *C. camphora* at 30°C, the nanoparticles are produced within 1 h, although completion of the reaction occurs in 118 h [64]. The absorption spectrum of the reduced product containing different quantities of the leaf extract has revealed that there are two absorption peaks, a strong peak at 440 nm due to particles of one shape in abundance and a weak peak at 360 nm owing to some scattered particles of different shape. It is apparent from the scanning electron microscopy (SEM) and transmission electron microscopy (TEM) images of silver nanoparticles that the morphology of the crystals are slightly different, although their size ranges between 55- and 80 nm. The nanocrystals produced from small quantity of the biomass are scattered and are of better quality. When the quantity of biomass is increased, the time of formation of nanocrystals is drastically reduced from 118 h for 0.5 g biomass to 24 h for 1.0 g [64]. However, in such cases, the nanoparticles are aggregated, while with low quantity of the biomass, they remain segregated. It has also been observed that with increasing biomass the shape of nanocrystals also changes. The different absorption maxima correspond to different types of the nanocrystals formed. It has been reported by Huang et al. [64] that *C. camphora* leaf contains alkaloids, hydroxybenzenes, anthracene, steroids, terpenoids, coumarins, lactones, linalools, polysaccharides, amino acids and proteins. The silver and gold nanocrystals have been produced from the dried biomass of leaves. The study of the Fourier transform infrared (FTIR) spectrum of the dried leaf biomass before and after reduction of Ag^+^ and Au^3+^ shows changes in the functional groups of biomolecules [64]. There appear absorption bands at 1,109, 1,631 and 1,726 cm^-1^ which are attributed to CO, C = C and C = O stretching frequencies, respectively, in the free leaf powder. The IR spectrum of the leaf biomass recorded after the reduction of silver/gold ions shows the disappearance of the band at 1,109 cm^-1^ assigned to polyols present in the leaf biomass. It has been concluded that polyols are mainly responsible for the bioreduction of metal ions leaving behind RCO, which in turn, may react with the solvent to give a neutral species. The decoction of the leaf is a mixture of many compounds which cannot be identified; nevertheless, some of the frequencies remained unaltered which is believed to be due to C = C or ring vibrations. Huang et al. [64] have suggested that the shape of nanocrystals is mainly due to the protective and reductive biomolecules in the suspension. This idea of protective and reductive biomolecules is conceptually vague because when the nanocrystals are separated and dried they do not contain biomolecules to stabilize them. The biomolecules in our opinion react with other species to stay as neutral molecules after the nanocrystals have been isolated from the solvent.

Development and regeneration of root/shoot can occur in IBA-mediated adventitious root in the presence of 100 to 250 μm Na_2_S_2_O_3_ in agar gel [65]. The authors claimed that the potential of Na_2_S_2_O_3_ in facilitating culture development has not been recognized prior to this report. Many experiments were performed with different agar gels where precipitation of silver ions occurs. Generally, the incubated plant tissue culture produce ethylene and accumulation of hormone occurs which does not favour the culture growth. Addition of Ag^+^ ions inhibits the ethylene action. Though no one has commented on the mechanism of action of Ag^+^ with ethylene, it is for sure that ethylene reacts with Ag^+^ to give stable complex. The evolution of ethylene is not inhibited rather ethylene forms silver complex as (C_2_H_4_) Ag. Merril et al. [66] and Costa-Coquelard et al. [67] have suggested that Ag^+^ is precipitated as colloidal AgCl which changes colour when exposed to sunlight. Further, they have suggested that the change in colour of AgCl is a function of nanoparticle size and chemical composition. It should be viewed with caution that the composition of AgCl does not vary and being aggregate it settles at the bottom of the container. This is true that reduction of Ag^+^ ion is hindered unless there is some reducing agent in that medium. The effect of AgNO_3_ and Ag_2_S_2_O_3_ on shoot and root growth is comparable, although in this work [65], Ag_2_S_2_O_3_ has not been directly used. Na_2_S_2_O_3_ was added to AgNO_3_ as a consequence of which Ag_2_S_2_O_3_ would have been formed according to the following equation:

$$2{\mathrm{AgNO}}_{3}+{\mathrm{Na}}_{2}S_{2}O_{3}\to {\mathrm{Ag}}_{2}S_{2}O_{3}+2{\mathrm{NaNO}}_{3}$$

The authors have examined the effect of thiosulfate ion on the root/shoot development but simultaneously ignored the effect of the nitrate ion and did not perform any experiment with free ion to exclude its impact. Many workers have quoted that [68-70] Ag^+^ ions react with polysaccharide, amino acids, protein, RNA and DNA to form nanoparticles. It is not always true because Ag^+^ may form complex with such electron donors, and for reduction, a reducing agent is required which in turn will be oxidized.

$${\mathrm{Ag}}^{+}+2{\mathrm{NRH}}_{2}\to {\left[\mathrm{Ag}{\left({\mathrm{RNH}}_{2}\right)}_{2}\right]}^{+}\;\mathrm{Complex}\;\mathrm{formation}$$

$$2{\mathrm{Ag}}^{+}+H_{2}\to 2\mathrm{Ag}+2H^{+\;}\mathrm{Ag}-\mathrm{nanoparticle}$$

Precipitation of Ag^+^ as AgCl in agar gel medium occurs due to the presence of HCl as a contaminant. If an excess of AgNO_3_ is added to this broth, only then free Ag^+^ ion will be available which may be reduced to nanosized particles. However, contrary to the present report, both the AgNO_3_ and Ag_2_S_2_O_3_ will furnish Ag^+^ ions which will have the same influence on the root growth, if the effect of ${\text{NO}}_{3}^{-}$ and $S_{2}O_{3}^{2-}$ ions is ignored [71]. In this work [65], the Ag_2_S_2_O_3_ was prepared by mixing 0.1 M solutions of AgNO_3_ and Na_2_S_2_O_3_ in 1:4 M ratio at ambient temperature. Since, according to the simple metathetical reaction as given below, the two components react in 2:1 M ratio, there is always an excess of Na_2_S_2_O_3_ in this preparation.

$$2{\mathrm{AgNO}}_{3}+{\mathrm{Na}}_{2}S_{2}O_{3}\to {\mathrm{Ag}}_{2}S_{2}O_{3}+2{\mathrm{NaNO}}_{3}$$

Silver nanoparticles may be present with large crystal (three to five times) of Na_2_S_2_O_3_ and hence the influence of $S_{2}O_{3}^{2-}$ ions on the shoot growth may be ignored. The development of root by Ag^+^ ion (obtained from AgNO_3_) in the presence of Cl^-^ ion is shown, which was obtained from Ag_2_S_2_O_3 _[65]. It is to be made clear that if the chloride ion is present in the solution, the entire AgNO_3_ will be precipitated and no free Ag^+^ ion will be available to exhibit its influence on root growth. If AgNO_3_ is in large excess and there is only little Cl^-^ ion available, some of it will be available as free ions.

$${\mathrm{AgNO}}_{3}+{\mathrm{Cl}}^{-}\to \mathrm{AgCl}+{{\mathrm{NO}}_{3}}^{-}$$

The silver ions may be available for interaction with other molecules. However, it is important to note that when AgNO_3_ is taken in the presence of Na_2_S_2_O_3_, the Ag_2_S_2_O_3_ thus formed remains dissolved, and both the Ag^+^ and $S_{2}O_{3}^{2-}$ ions are available.

$$2{\mathrm{AgNO}}_{3}+{\mathrm{Na}}_{2}S_{2}O_{3}\to {\mathrm{Ag}}_{2}S_{2}O_{3}+2{\mathrm{NaNO}}_{3}$$

$${\mathrm{Ag}}_{2}S_{2}O_{3}⇌2{\mathrm{Ag}}^{+}+S_{2}{O_{3}}^{2-}$$

The cumulative effect of both the Ag^+^ and $S_{2}O_{3}^{2-}$ ions on root development may be encountered. To eliminate the effect of $S_{2}O_{3}^{2-}$ ion, similar experiment, only with Na_2_S_2_O_3_ mediated with IBA showed that the concentration of Na_2_S_2_O_3_ above 100 μm was most effective [65].

Song and Kim [21] have reported the synthesis of silver nanoparticles using the leaf extract of five different plants, namely pine, persimmon, ginkgo, magnolia and platanus. Of all the five leaf extracts, magnolia leaf broth was found to be the most effective reductant for silver nitrate to silver nanoparticles. The process of production of nanoparticles was so fast that nearly 90% of Ag^+^ ion was converted to silver metal in about 11 min at 95°C. The average particle size ranges between 15- and 500 nm. The authors have observed that the size of the particles can be monitored by (i) changing the temperature and (ii) the concentration of AgNO_3_ and (iii) that of the leaf extract. It has already been studied that the particle size of the nanocrystal decreases with the increase in reaction temperature. Song and Kim [21] have hypothesized that with increasing temperature the rate of reduction of Ag^+^ ion to Ag also increases, stopping the secondary reduction process on the surface. It is worth observing that there is no secondary reduction process as the Ag^+^ ion requires only one electron (Ag^+^ + *e*^-^ → Ag) for reduction to metal. The secondary reduction will mean capturing one more electron by silver atom to become Ag^-^ which is impossible because it cannot hold an extra electron into its orbit.

There are some vascular plants which store crystal metal and are called metallophytes, for instance, *Brassica juncea*, *Medicago sativa*, etc. They accumulate metal up to 13.6% weight in 72 h when it is available for absorption in the form of salt, like AgNO_3 _[72]. It is quite obvious that reduction of AgNO_3_ is followed by absorption which means that the plant contains some compounds which reduce Ag^+^ to Ag nanoparticles of approximately 50 nm size. It has been demonstrated that the metals thus stored in the plants as nanocrystals are analytically pure to the lowest limit of detection by any instrument like AAS. The sequestering of metal by plant from a large heap of sand, sediments and non-essential non-metals is a process that saves time and manpower. If bacteria and small plants are grown in such mining areas where a large heap of nanocrystal of metal ions is available, they can be easily taken up by them and harvested. The extraction of metal by conventional method is a tedious task as it takes a long span of time; even then, it is not as pure as sequestered by plants. It has been reported by Blaylock et al. [73] that the addition of a chelating agent like ethylene diamine tetraacetate (EDTA) to the soil increases the bioavailability of the metal. It is true that EDTA forms a soluble complex with metal ions available but not the metal. The EDTA therefore acts as a carrier, not as a reductant. Since EDTA is not a selective chelating agent, it may hook up all metal ions regardless of their useful/harmful effect. If the metal remains bound to a chelating agent, it is not available even to the plants and hence may cause a deficiency of certain essential trace metals in them.

Haverkamp and Marshall [74] have studied the uptake of AgNO_3_, Na_3_Ag(S_2_O_3_)_2_, Ag(NH_3_)_2_NO_3_ and their reduction to nanoparticles by *B. juncea*. Quantitative determination of Ag by AAS and XANES has been done. The reduction of metal depends on the chemicals present in the plant and the concentration of metal salts in the solution. Gold [75-77], silver [78,79], copper [80] and gold-silver-copper alloy [81] nanoparticles have been reported to be present in the plants. Besides the plants, some microorganisms also induce the metal ions which are accumulated and translocated in different parts of the plants. Ni, Cu, Cd, Pb and Cr have not been exclusively found to yield nanoparticles, perhaps these are also not common metals required by the plants for their growth. The uptake and distribution of metal ion/metal itself in the plant is a matter of debate. It is not clear whether nanocrystals are formed outside of the plants and then transported through the membrane into various parts or if the nanoparticles are formed within the plant by the reduction of the metal salt. Some workers [76,77] believe that nanoparticles may be formed on the root tips and then transported into plant. If the root exudes some organic molecules which may reduce the metal salts, only then metal nanoparticles may be formed and transported. Since the root absorbs the minerals dissolved in water by osmotic pressure or capillary action, the metal salts ascend in ionic form and subsequently reduced to elemental form as nanoparticles [82]. The rate of growth of silver nanoparticle is independent of the concentration of salt but mobility is dependent on the size of ion. If the Na_3_Ag(S_2_O_3_)_2_ and AgNO_3_ are taken, the availability of Ag^+^ ion in AgNO_3_ will be larger than the $\text{Ag}{\left(S_{2}O_{3}\right)}_{2}^{3-}$ ion. The authors suggest that three forms of Ag appear to be present (Ag^+^, AgNO_3_ and Ag_2_O). It is not the form of Ag but the anion in equilibrium with the cation, ${\mathrm{AgNO}}_{3}\to {\mathrm{Ag}}^{+}+{\mathrm{NO}}_{3}^{-}$. However, the rate of deposition of Ag nanoparticle from AgNO_3_ containing small anion is faster than that with large anion like $S_{2}O_{3}^{2-}$.

#### ***Gold nanoparticles***

Biosynthesis of gold nanoparticles depends on the (i) concentration of plant extract or biomass, (ii) concentration of metal salt, (iii) temperature and (iv) pH of the solution. It has been observed during the synthesis of gold nanoparticles by *Avena sativa* biomass that several types of nanoparticles are produced with different structures [83]. The face centred cubic, tetrahedral, hexagonal, decahedral, icosahedral and irregular rod-shaped gold nanoparticles were produced. The yield was highest at pH 3. At higher pH, the nanoparticles of small size are produced. However, rod-shaped nanoparticles were produced at all pH which have been reported to be formed mainly by electrodeposition. In the present case, KAuCl_4_ was taken as the source which on dissolution in water gives ${\mathrm{AuCl}}_{4}^{-}$ anion. It ought to be bonded to carboxylic groups which are already protonated at low pH. The oat biomass shows the ability to bind ${\mathrm{AuCl}}_{4}^{-}$ and its subsequent reduction to gold nanoparticles. They have been produced from dead and live tissue of alfalfa [76,84-86], hops [87], fungus [88,89] and algae [90-92]. The basic idea behind the formation of nanoparticles is the reduction of metal ion to elemental metal. The plant biomass or even the extract of green leaves must, therefore, contain such chemicals so as to reduce the metal ion. As mentioned earlier, the plants which have aroma contain flavonoids, reducing sugars or alcohols/phenols which act as reductant leading to the formation of nanoparticles. The focal point of our attention must therefore be directed towards all species and smelling leaves, flowers and plants for the synthesis of nanoparticles because they all contain such chemicals which reduce the metal ion to metal nanoparticles. The FTIR spectra of leaf extract or dried leaf biomass, before and after the formation of nanoparticles, reveal the changes in the functional groups. It shows the presence of OCH_3_ group in *Phyllanthin* extract [93] eugenol in clove extract [94] and polyol in *C. camphora* leaf [64]. Geranium leaf, neem leaf [95,96], lemon grass, etc. have been used to produce gold nanoparticles [97]. As the progress is made in nanotechnology, biosynthesis is made easy. Instead of using the aqueous extract of plant leaf by boiling, only sun-dried leaf powder in water at ambient temperature is now used. In such procedure, a moderator and accelerator like ammonia is not needed, but the concentration of leaf extract is the rate-determining step. It is a significant step in bioreduction of chloroaurate ions [AuCl_4_]^-^ that biomolecules of molecular weight less than 3 kDa can cause its reduction.

The metals can be sequestered from a mixture of several metals in different forms such as oxides, halides, carbonates, nitrates, sulphates, acetate, etc. Zhan et al. [98] have reported the biosynthesis of gold nanoparticles by *Cacumen platycladi* leaf extract. They have made a simulation of the active components and prepared a mixture of several known chemical substances on the basis of FTIR spectral data of *C. platycladi* leaf extract before and after the biosynthesis of nanoparticles. They were characterized by UV-visible (UV-vis) spectroscopy, thermogravimetric analysis (TGA), X-ray diffractometry (XRD), SEM and TEM. The structure, shape, temperature, pH and distribution of nanoparticles were studied. The extract was found to contain polysaccharide, reducing sugar, flavonoid and protein. The addition of *C. platycladi* leaf extract to aqueous solution of HAuCl_4_ showed a change in colour from pale yellow to brownish red in a span of 5 min. Its UV-vis spectrum exhibited *λ* max at 530 nm, the intensity of which increased with time and attained a maximum after 90 min showing the completion of the reaction. Surprisingly, the average nanoparticle size is fairly small, of the order of 15.3 nm. The FTIR spectrum after nanoparticle formation showed a reduction in the intensity of some prominent bands. The IR spectrum of purified nanoparticles showed the reduction of peaks at 3,448, 1,610 and 1,384 cm^-1^ which means that some of the leaf biomass remains stuck to nanoparticles; otherwise, elemental gold would not show any peak in the IR spectrum. The TGA and differential thermal analysis (DTA) results of the gold nanoparticles after thorough washing were recorded. It starts decomposing after 100°C and completes at 525°C; thereafter, a plateau appears which remains stable even at 800°C. The metal thus left as residue is actually gold oxide because the TGA was done in open where oxidation of metal may not be avoided. The authors have not clarified whether the end product is pure metal or metal oxide. The DTA of course shows two distinct changes in temperature (234°C and 507°C) indicating volatilization of organic components from leaf extract which may have acted as stabilizer or protective substance. Phenols, in fact, act as reducing agent and they themselves get oxidized to quinone. This property should have been discussed at length.

Random use of any metal nanoparticles in plants or food crops may not produce desired vegetative growth or enhance the yield of food crops. It must be known which trace elements are useful for the plant under experiment so that the same nanoparticles are used to increase the yield. The *B. juncea* seedlings on treatment with gold nanoparticles in the field (foliar spray) showed changes both in growth and yield of seed [99]. Like CuO nanoparticle in wheat [100], gold nanoparticle was also accumulated in *Brassica *[99]. The percentage of germination increased when *B. juncea* seedling were sprayed/inoculated with 25-ppm gold nanoparticles. However, as the concentration of gold nanoparticles increases, the rate of germination is slowed down. The authors have suggested that the antagonistic effect of gold nanoparticles slows down the effect of ethylene; as a result of which, an increase in the number of leaves of *B. juncea* occurs. In fact, it is not the antagonism of gold nanoparticles but the complexation of ethylene with gold or adsorption of ethylene on gold nanoparticles. An average 19% increase in the seed of *B. juncea* was noted after treating the plant with about 10-ppm gold nanoparticles. However, it is not economically feasible as the cost of gold nanoparticles (10 mg L^-1^) sprayed seems greater than the yield of the crop nevertheless; it is an attempt towards a bright future for increased food crop produced with engineered gold nanoparticles.

#### ***Nickel, platinum and palladium nanoparticles***

Bali et al. [101] have studied the formation of platinum nanoparticles from Pt(II) by *M. sativa* and *B. juncea* plant biomass. The conversion of Pt(II) to metallic platinum was studied in acidic medium between pH 2 and 3. However, such high pH amongst plant kingdom is never achieved. This process can be used to extract metals from clinical disposal sites to prevent recycling in the soil. Generally, the metals in the soil or at mining sites exist in the form of salts rather than a co-reduction compound. The platinum metal concentration in this study showed the accumulation of platinum between 0.77 and 36.83 mg of platinum per gram of dry biomass of *M. sativa*. Spherical-shaped palladium nanoparticles have also been obtained using peel extract of *Annona squamosa *[102]. It is a useful study of platinum metal uptake by plants which can be extended to other metal ions of this group of metals, *viz*. Ni, Pt and Pd. Both the living and dead organisms are equally useful in producing nanosized crystal of metal [103]. Reduction of Pd(II) to elemental palladium has been achieved by formate or hydrogen [104].

### Beneficial and adverse effects of metal nanoparticles

**Figure 5:**
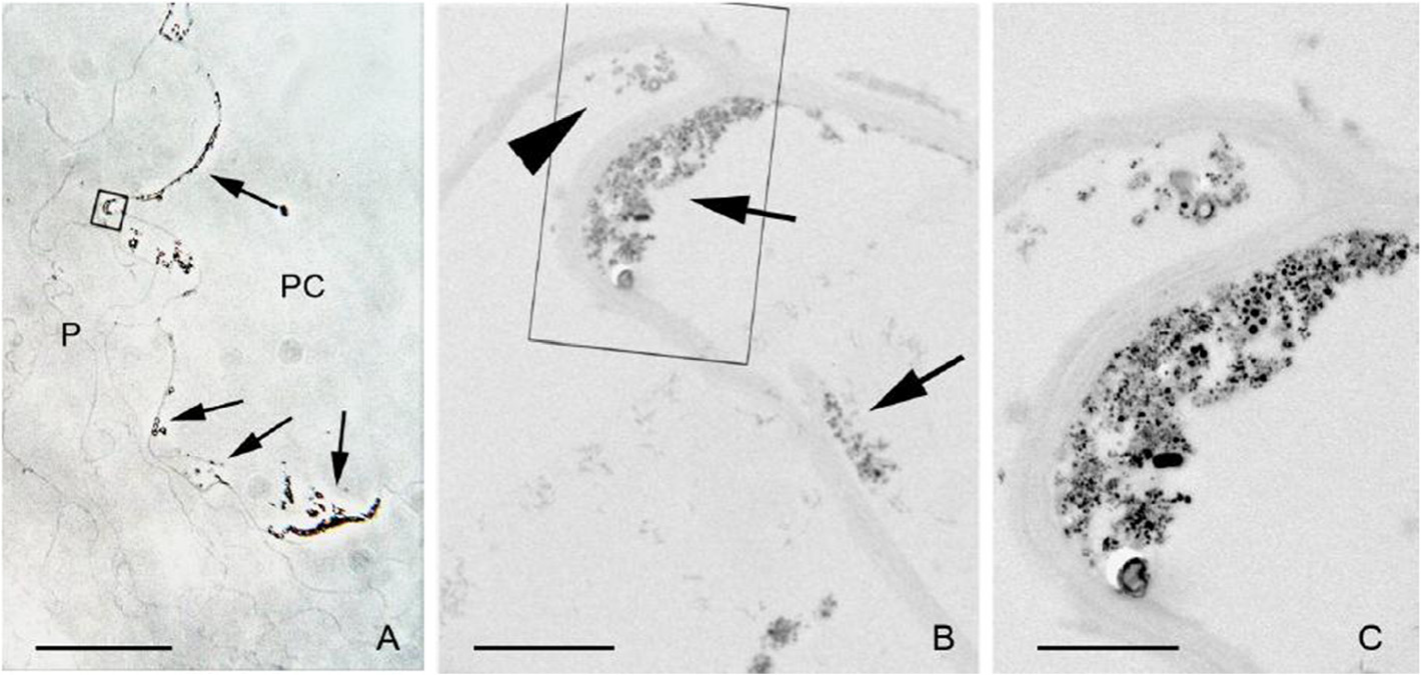
**Penetration of nanoparticles into the first cell layer surrounding the pith cavity. (A)** Phase contrast image of the parenchymatic cells (P) closer to the pith cavity (PC). The nanoparticle aggregates on the application surface appear as an optically dense material (arrows). **(B)** Transmission electron micrograph of the region squared in (A). Nanoparticle aggregates appear in the cell wall facing the pith cavity (arrows) and into the cytoplasm of the first cell layer (arrow head). **(C)** High magnification of the region squared in (B). The intracellular aggregate is smaller than the extracellular one in the pith cavity. Bar in (A) = 40 μm, (B) = 2 μm, (C) = 1 μm [105].

Nanoparticles of specific size are capable of penetrating and migrating to different regions of plant cells [105]. These nanoparticles can be stopped at certain point or their movement may be accelerated by the use of small magnets provided that the nanoparticle is magnetic in nature as the non-transition metal ions are not attracted towards a magnet. Some experiments using carbon-coated nanoparticles of iron have been shown to have some unusual influence on the plant growth and yield of the grain or fruit. The nanoparticle movement may be directed to certain parts of the plant or certain specific organ in microbes/animals. The disease in plant or animal may thus be effectively treated with nanoparticles [106]. Corredor et al. [105] have shown the application of carbon-coated iron nanoparticle to pumpkin plant for the dissected release of chemicals into the specific part of the plant prone to infection by pathogens. The nanoparticles enter the living cells and are distributed over the entire part, the mechanism of which is yet to be understood. The nanoparticles were applied in different modes, namely by injection and spraying. Though a very small quantity of nanoparticles is required for injection, it is practically not possible on large scale and hence, generally, spraying is done. Sometimes, small magnets are inserted at certain points of the plant so that immobilized nanoparticles are accelerated at target point. The dark precipitate deposited in the inner surface of the pith cavity is visible even with the naked eye (Figure 5). The presence of nanoparticles was confirmed by SEM and TEM images. These nanoparticles appeared as intracellular aggregates and have also been observed in the cytoplasm of epidermal cells. Plant cells respond to a high density of nanoparticles by changing their subcellular organizations. The number of nanoparticles and cytotoxicity are related to each other. Nanoparticles when sprayed normally penetrate through the stomata and so are used for pathogens of different species. They may therefore be killed by nanoparticles preventing the plant/fruit from further damage.

Of the various nanoparticles, gold nanoparticle has assumed more importance due to its application in almost all areas of medicine [107-109] and technology. Recently, the gold nanoparticles synthesized from *Gnidia glauca* flower extract has been used as chemocatalytic agent in the reduction of 4-nitrophenol to 4-aminophenol in the presence of sodium borohydride [110].

**Figure 6:**
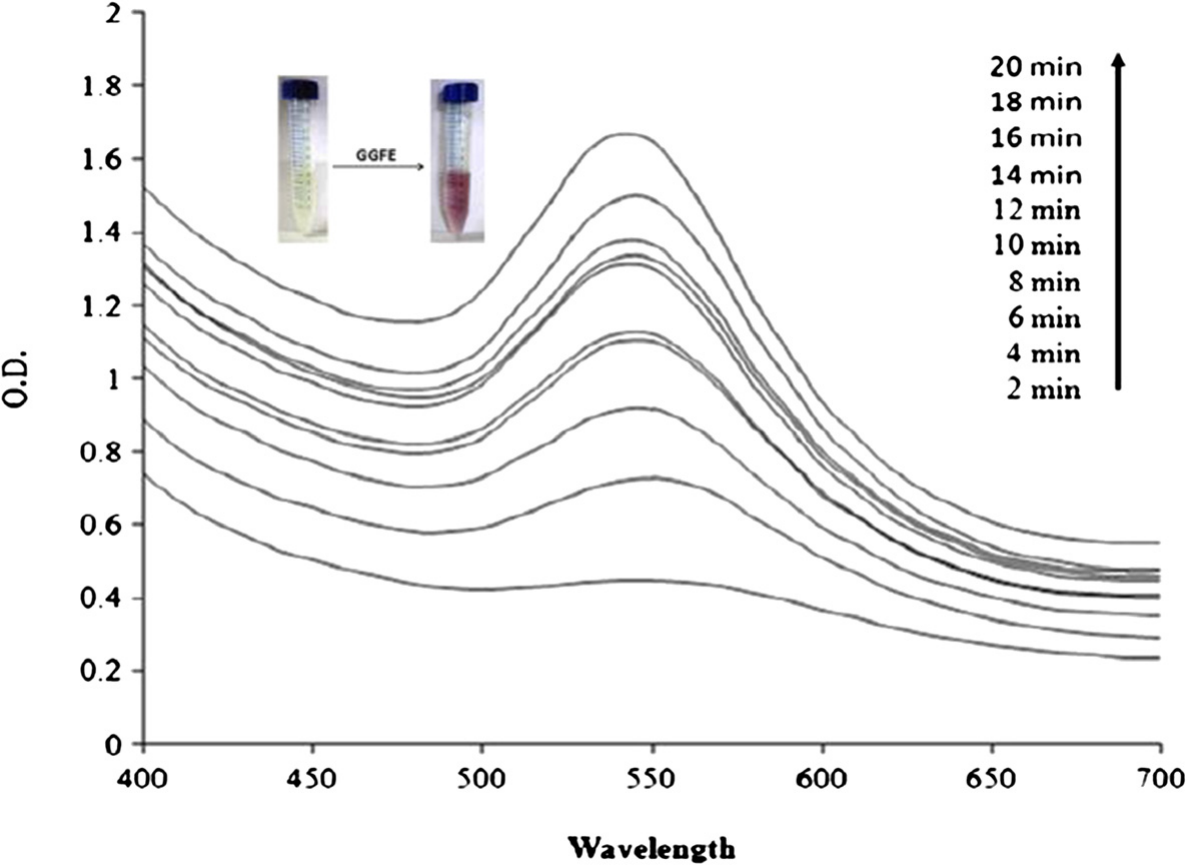
**Time course of gold nanoparticle formation.** As obtained with different concentrations of chloroauric acid using *Gnidia glauca* flower extract at 40°C [110].

The formation of nanoparticles may be followed spectrophotometrically by the change in colour from yellow to dark red in the visible region of the spectrum between 450- and 600 nm (Figure 6). The UV-vis spectrum of gold nanoparticles as a function of time shows that the reaction is completed within 20 min. It has been shown that the formation of gold nanoparticles starts 2 min after the interaction of plant extract with HAuCl_4 _[110]. The current method [110] of gold nanoparticle synthesis is faster and efficient than that reported earlier by Vankar and Bajpai [111] which took approximately 2 h for the completion of reaction. At concentration as low as 0.7 mM, the synthesis was optimum, and above this concentration, the formation of gold nanoparticles ceases to continue (Figure 6). The rate of synthesis of gold nanoparticles from *G. glauca* flower extract increases with increasing temperature and attains maximum between 40°C and 50°C. A similar pattern was found to follow when gold nanoparticle was synthesized from *Nyctanthes arbortristis* flower extract [112]. In this case, the particles are spherical in size ranging between 5- and 20 nm [113,114]. Polydispersed gold nanoparticles can be obtained from *Rosa hybrida* petal extract [115]. When the concentration of HAuCl_4_ is low, gold nanoparticles of smaller size are produced, although they are often covered with larger particles as aggregates [114]. The FTIR spectra of dried *G. glauca* flower [110] extract before and after the synthesis of nanoparticles revealed a decrease in all stretching frequencies of the probable functional groups of the phenols, flavonoids and amines present in the extract. It suggests a decrease in the concentration of the functional groups after the synthesis of gold nanoparticles, which is obvious. During the phytosynthesis of metal nanoparticles, all alcohol, aldehyde and phenol present in the plant extract are oxidized (as shown below), and the metal ions are reduced to metal nanoparticles:

Alcohol → Aldehyde

Aldehyde → Carboxylic acid

Phenol → Ketone

Flavonoids → Flavone

These nanoparticles may be used as chemocatalytic agent in the reduction and degradation of organic compounds. Photocatalytic degradation of methylene blue was done under sunlight by the silver nanoparticles synthesized from *Morinda tinctoria* leaf extract. The deep blue colour of the dye starts fading after 1 h with the above experimental conditions under sunlight. The maximum absorbance for methylene is at 660 nm. The colour of methylene blue turned light green after 1 h and finally became colourless after 72 h showing its degradation up to a maximum of 95%. This demonstrates the photocatalytic activity of silver nanoparticles for methylene blue which may be exploited for the benign treatment of dye stuffs [116]. Ganaie et al. [117] have explored the catalytic degradation of dye such as Alizarin Red S and Remazol Brilliant Blue R by silver nanoparticles in the presence of hydrogen peroxide and sodium borohydride, respectively. The degradation of dye, as pollutant, was found to increase rapidly which was monitored spectrophotometrically by the decrease in absorbance. A variety of methods have been developed to synthesize the metal nanoparticles, although the shape and size vary greatly with the concentration of precursor metal and the reductant. The silver nanoparticles prepared from *Citrullus colocynthis* extract were found to be spherical in shape and approximately of 31 nm with different morphology [118]. The use of silver nanoparticles in medicine prompts scientists to explore more application in this area [119]. Biological methods for the synthesis of nanoparticles such as using microorganism [120], enzymes [121] or plant extract [21] are eco-friendly and efficient. Satyavani et al. [118] have recently studied the cytotoxicity of silver nanoparticles against cancer cell lines *in vitro*. The nanoparticles showed a decrease in viability of the HEp2 cells. The effect is time and concentration dependent. When the cancer cells were exposed to 50 nM concentration of silver nanoparticles for 5 h, their viability was reduced to 50% which is considered as IC_50_. The longer the exposure time, the greater the toxicity. The silver nanoparticles possess angiogenic properties [121], and therefore, it can be tested against various types of cancer cells. The effect of silver nanoparticles on osteoblast cancer cells has also been studied. It has been shown that a single dose of as little as 3.42 μg mL^-1^ of IC_50_ is more effective than the toxic heavy metals [122]. The replication of cancer cells under experimental conditions is inhibited regardless of the method of synthesis of silver nanoparticles. The release of lactate dehydrogenase is a marker of the effect of silver nanoparticles on cancer cell, which is significantly increased compared to untreated cells. It has been nicely demonstrated that silver nanoparticles caused death of cells through apoptosis which was also shown by cellular DNA fragmentation. The HEp2 cells treated with silver nanoparticles showed the cleavage of double strand of DNA fragment. It was observed that silver nanoparticles are manifold more effective against HEp2 cancer cells than silver ion [118], although the mobility of silver ion is obviously greater than the silver atom. The cytotoxicity of silver nanoparticle is mainly due to its interaction with the functional groups of the proteins within the cancer cell and nitrogen bases in DNA. It has been reported that green tea and decaffeinated green tea also inhibit activity of H1299 human lung carcinoma cell line. It is believed that its activity is synergized by polyphenols. Since such metal nanoparticles are not selective, they may equally damage the living cells. The living cells have the ability to repair themselves even though they may also be prevented from damage by such metals while treating for cancer.

In a study, Patil et al. [123] have synthesized silver nanoparticles from *Pergularia daemia* latex. They characterized and studied its toxic effect on some mosquitoes and non-target fish. Such studies are not common [123,124] even though an attempt has been made to see the toxicity of metal nanoparticles. The importance of such studies lies in its benign effect on the environment. Silver nanoparticles are also synthesized by dry and fresh latex of *P. daemia*, but the yield of nanoparticles by fresh latex was larger than that synthesized by dry latex. A comparison of both types of silver nanoparticles was made; an absorption spectrum showed a peak at 520 nm which is generally the characteristic of silver nanoparticles formed along with some of the biomolecules present in the latex or extract. Richardson et al. [125] have shown that plant extract containing carbohydrates and proteins serve as reducing agent for silver ions. Quercetin, a flavone derivative, was shown to be involved in the formation of silver nanoparticles [126], perhaps by catalysing the reaction through dissolved oxygen in the solutions. *Jatropha curcas* latex is known to reduce Ag^+^ to very small size nanoparticles of the order 20- to 30 nm. This plant is known to contain a peptide called curcacycline A and B which is involved in the reduction and stabilization of silver nanoparticles [127]. In the case of *P. daemia* latex, the protein part seems to be responsible for the synthesis of silver nanoparticles. The nanoparticles laced with latex are toxic to mosquito larvae, and in short-term experiment, it may be useful. However, contradictory report has also appeared that silver nanoparticles induce embryonic injuries and reduce survival of zebra fish [128]. The ability of silver nanoparticles as toxic material to reduce pathogens without disturbing the benign microbes and fish should be viewed with caution. Long-term study can only prove if it may be safely used without disturbing the ecosystem.

### Metal oxide nanoparticles

Numerous positive effects of engineered metal oxide nanoparticles have been practically proved (Table 2). It has been observed that SiO_2_ and TiO_2_ nanoparticles in appropriate ratio increase nitrate reductase activity in soybean, increase its capacity to absorb fertilizer and eventually reduce the time for germination [129]. They also enhance the rate of photosynthesis in spinach [130,131]. It is worth noting that nano-Al_2_O_3_ inhibits the root growth in maize and cucumbers. This seems as if the nanoparticles of certain elements may have adverse effect on plants or even in man [132]. The effect of silver and titanium dioxide nanoparticles on the growth inhibition of aquatic plants has been studied by Kim et al. [133]. Since the size and structure of nanoparticles have different properties from their salt or bulk material, they drastically alter or modify the physicochemical properties [134,135]. Natural availability of Ag and TiO_2_ nanoparticles makes them prominent. Major work has been done with TiO_2_ as it is a naturally occurring mineral which is capable of existing as rutile, anatase, brookite and amorphous forms [136]. TiO_2_ can generate potential reactive oxygen species (ROS) at its surface, in the presence of UV light [137], though ROS activity has been shown even in the absence of light [138]. Lethal effect of silver nanoparticles on bacteria [139] and yeast [52] are known [53,140]. Photocatalytic degradation of indigo carmine by TiO_2_-strewn sheet under UV light as a function of time has been studied. It has also been investigated spectrophotometrically. The concentration of indigo carmine dye after photodegradation was analysed at its absorption maximum at 610 nm. The intensity of this peak decreases with the passage of time eventually reaching the baseline indicating the complete degradation after about 5 h [141]. Since metal oxide nanoparticles, such as ZnO, MgO, TiO_2_ and SiO_2_, are also known to possess antimicrobial activities, they can be exploited in the treatment of common bacterial infection and in the sterilization of surgical instruments, but their toxicity to biological systems may be overlooked [142]. Enhanced antibacterial activity of *Argemone mexicana* treated with iron oxide nanoparticles was also reported against *Proteus mirabilis* and *Escherichia coli *[143]. The silver ions are also effective against these microbes, but the efficiency depends on its microlevel concentration [144]. It was found that *Lemna paucicostata* (7-day-old) grown in the presence of different concentrations of Ag and TiO_2_ nanoparticles inhibited its growth [133]. At ≥1 ppm, silver nanoparticles showed significant decrease in *L. paucicostata* growth, but with nanoparticles ≤100 ppm, the growth is completely inhibited. On the contrary, the growth inhibition by TiO_2_ nanoparticles is effective only at 500-ppm level. These nanoparticles may be used to eradicate the unwanted aquatic weed and plants, but the damage to other plants and aquatic animals may not be prevented. It can work in isolated system, but in ponds, it may cause havoc by destroying the non-target plants and animals like fish, etc. Crop yield and grain quality may be improved by the use of manufactured nanomaterial. The method of application and absorption may vary; the manufactured nanomaterial may be sprayed or mixed with the soil. Experiment with nano-CeO and nano-ZnO on soybean showed an increase in quality and yield of crops. The ZnO nanoparticle was taken up by the plant and distributed uniformly throughout the plant tissues. All manufactured nanomaterials may not be equally effective for all crops. In this case [145], the soybean treated with CeO_2_ gave unexpected result. The nano-CeO_2_-treated plants had decreased leaf counts irrespective of its concentration. Even the lowest concentration showed retarded growth in the harvested plant. The stunted plants may be grown with CeO_2_ nanoparticles, but any increase in crop yield has not been recorded.

**Table 2:** Effects of engineered metal oxide nanoparticles on plants

**Nanoparticle**	**Size (nm)**	**Plant**	**Concentration**	**Effect**	**References**
Al_2_O_3_		Corn, cucumber, lettuce, radish, rapeseed, ryegrass	2,000 mg L^-1^	No effect on germination	[44]
	13	Carrots, cabbage, cucumber, maize	2,000 mg L^-1^	Reduced root growth	[146]
		Corn	2,000 mg L^-1^	Reduced root length	[44]
CeO_2_	7	Alfalfa	1,000 and 2,000 mg L^-1^	Slightly reduced shoot growth	[147]
		Tomato	2,000 mg L^-1^	Reduced shoot growth	[147]
		Cucumber	2,000 mg L^-1^	Reduced shoot growth	[147]
		Maize	500, 1,000 and 2,000 mg L^-1^	Reduced shoot growth	[147]
		Alfalfa	500 mg L^-1^	Reduced biomass	[147]
		Maize	500 to 2,000 mg L^-1^	Reduced germination	[147]
		Maize	4,000 mg L^-1^	Reduced root growth	[147]
		Tomato, cucumber	2,000 mg L^-1^	Reduced germination	[147]
		Tomato	1,000 to 2,000 mg L^-1^	Reduced root growth	[147]
		Alfalfa	2,000 to 4,000 mg L^-1^	Reduced root growth	[147]
		Soybean	2,000 mg L^-1^	Reduced germination	[147]
	7	Alfalfa, corn, soybean	500, 1,000, 2,000 and 4,000 mg L^-1^	Increased root and stem growth	[147]
	<25	Wheat	100 mg L^-1^		[148]
	8.0 ± 1.0	Coriander	125 mg kg^-1^	Increased shoot and root length, increased biomass, increased catalase activity in shoots and increased ascorbate peroxidise activity in roots	[149]
	231 ± 16	Rice	62.50 and 125 mg L^-1^	Reduced H_2_O_2_ generation in shoots and roots	[150]
			500 mg L^-1^	Increased electrolyte leakage and lipid peroxidation in shoots	[150]
FeO	10.2 ± 2.6	Clover	3.2 mg kg^-1^	Reduced aboveground and belowground biomass	[49]
Fe_3_O_4_	20	Pumpkin	500 mg L^-1^	No toxic effect	[29]
	7	Cucumber, lettuce	62, 100 and 116 mg L^-1^	Low to zero toxicity	[47]
Magnetite (iron oxide)		Soybean	0.2, 0.4, 1.0 and 2.0 mg L^-1^	Increased chlorophyll levels	[151]
Mixture of SiO_2_/TiO_2_		Soybean		Increased germination and shoot growth, increased nitrate reductase activity, increased absorption and utilization of water/fertilizer and enhanced antioxidant system	[129]
Ni(OH)_2_	8.7	Mesquite	2 mg L^-1^	No effect	[152]
Nanosized TiO_2_	21	Wheat	10 ppm	Reduced germination	[153]
			2 and 10 ppm	Increased shoot and seedling lengths	
			100 and 500 ppm	Reduced shoot and seedling lengths	
			100 ppm	Increased root dry matter production	
Nanoanatase (TiO_2_)	4 to 6	Spinach	0.25%	Enhanced rca mRNA expressions, protein levels, activity of Rubisco activase, Rubisco carboxylation, the rate of photosynthetic carbon reaction, single plant dry weight and chlorophyll content	[154]
	5	Spinach	0.25%	Improved spinach growth related to N_2_ fixation by TiO_2_	[155]
	5	Spinach	0.25%	Improved light absorbance, transformation from light energy to electron energy, and active chemical energy and promoted carbon dioxide assimilation	[156]
Rutile (TiO_2_)		Spinach (naturally aged)	0.25% to 4%	Increased germination and germination and vigour indices, plant dry weight, chlorophyll formation, ribulose bisphosphate carboxylase/oxygenase activity and photosynthetic rate	[10]
		Spinach	0.25% to 4%	Promoted photosynthesis, the rate of evolution of oxygen in the chloroplasts was accelerated	[130]
TiO_2_/inorganic bentonite clay	30/1 to 60	Maize	300 and 1,000 mg L^-1^	Inhibited hydraulic conductivity, leaf growth and transpiration	[157]
ZnO	8	Soybean	500 mg L^-1^	Increased root growth	[147]
	9 to 37 (mean 19 ± 7)	Ryegrass	1,000 mg L^-1^	Reduced biomass, shrank root tips, epidermis and root cap were broken, highly vacuolated and collapsed cortical cells	[44]
		Corn	2,000 mg L^-1^	Reduced germination	[44]
		Corn, cucumber, lettuce, radish, rapeseed, ryegrass	2,000 mg L^-1^	Reduced root growth and elongation	[44]
	5	Zucchini	1,000 mg L^-1^	Reduced biomass	[46]
	8	Soybean	2,000 and 4,000 mg L^-1^	Decreased root growth	[147]
3-Amino-functionalized SiO_2_		Lettuce	0.013% to 0.066% (*w*/*w*)	No effect on germination, improved shoot/root ratio	[13]

### Beneficial and adverse effects of metal oxide nanoparticles

Bulk and nanosized TiO_2_ particles have different impacts on plants and microorganisms. Concentrations of bulk and nanoparticles ranging from 1 to 500 ppm have been tried on wheat germination and seedling growth. The Ti compounds showed the following improvements after the crop or seedlings were treated with it [158]:

(i) The enhancement of yield of various crops, 10% to 20%

(ii) An improvement of some essential element contents in plants

(iii) An increase in enzyme activity like peroxide, catalase and nitrate reductase activity in plant tissue

(iv) Enhancement of chlorophyll pigment

TiO_2_ nanoparticles have also been demonstrated to increase the rate of germination and growth of spinach (*Spinacia oleracea*) [10]. It is believed that such nanoparticles influence the plant growth due to their antimicrobial properties. However, it is one of the several factors but not the consequence of antimicrobial properties that is responsible for the growth of plants. Nanosized TiO_2_ particles can promote nitrogen metabolism in the plant leading to growth as a whole. On the other hand, alumina nanoparticles affected adversely the elongation of corn, cucumber, soybean, cabbage and carrot [146]. Besides TiO_2_, other metal nanoparticles have also been shown to influence the crop production and their vegetative growth (Table 2). In almost all studies, the size of nanoparticles appears to be the critical factor. As the concentration of metal or metal oxide nanoparticles increases, the growth increases and reaches an optimum value after which either it becomes constant or retardation in growth occurs. In such instances, the enzyme activity is either lost or the nanoparticles block the passage of other nutrients as a consequence of accumulation. The germination time of seed with TiO_2_ was reduced to 0.89 days; shoot and seedling length was also increased after treatment of wheat seeds with TiO_2_ nanoparticles at 2- and 10-ppm concentration. When the concentration was raised to 100 ppm, no improvement was observed [10]. The effect of TiO_2_ nanoparticles on seed growth and germination is size and concentration dependent, because the small particles can easily penetrate the cell wall of the plant and move to various other parts. TiO_2_ nanoparticles coupled with SiO_2_ increase the nitrate reductase enzyme in soybean [129], increase the capacity to absorb water and fertilizer and promote its antioxidant activity, the germination of seed and growth of the plant.

Zhou et al. [100] have made a distinction between adsorption and absorption. Adsorption is a surface phenomenon, while absorption depends on the concentration, size factors and temperature. Both adsorption and absorption may occur simultaneously in plants [159]. The uptake of nanoparticles may be checked in plants, but adsorption is the accumulation of nanoparticles that remains on the surface of the plants. The adsorbed CuO nanoparticles on the root surface were checked in the presence of complexing agents such as Na_4_EDTA and NaOAC. It is however very interesting to believe that EDTA dissolves CuO nanoparticles by forming complex with released Cu^2+^.

$$2\mathrm{CuO}+{\mathrm{Na}}_{4}\mathrm{EDTA}\to {\mathrm{Cu}}_{2}\left(\mathrm{EDTA}\right)+2{\mathrm{Na}}_{2}O$$

According to this metathesis, free Cu^2+^ will not be available for subsequent reaction with EDTA, rather Na^+^ is replaced by Cu^2+^ ions leading to the formation of Cu_2_(EDTA). The equilibrium between CuO nanoparticles and Cu_2_(EDTA) depends on the quantum of Na_4_EDTA added and that of CuO nanoparticles present. Since the authors insist that the equilibrium between CuO nanoparticles and Cu^2+^ is lost, the dissolution of CuO nanoparticles is enhanced. It is not true because the number of moles of EDTA-Cu complex produced will correspond to the number of moles of EDTA added. The speculation that Cu nanoparticles adhered to the root is only due to complex formation may not be true, as there must be some complexing agent exuded by the root hairs. The adsorption of CuO nanoparticles by wheat root is concentration dependent. The authors have unnecessarily compared the adsorption with the uptake of nanoparticles [100]. The amount of nanoparticles adsorbed is actually retained on the surface due to electrostatic force, and fewer particles are absorbed into the plant system. When CuO nanoparticles are adhered to the outer surface of the root, they may not be transported to the cells unless they are absorbed. The absorption and uptake are synonymous in the present context because wherever it is absorbed it is in fact taken up by the plant. The authors have concluded that Na_4_EDTA increases the solubility of CuO nanoparticles, if it is the case, a mixture of CuO nanoparticles and Na_4_EDTA should be administered to the plant instead of taking the troublesome route of adherence of nanoparticles and their subsequent dissolution by Na_4_EDTA for absorption.

Contradictory reports have been received on the application of CuO nanoparticles on plants. While CuO nanoparticles have been shown to absorb in wheat, it has been reported to produce adverse effect on maize plants [160]. It has been reported that CuO nanoparticles have apparently no effect on the germination of maize seeds; nevertheless, it increased chlorosis and inhibited the growth of maize seedlings when exposed to 100 mg L^-1^ CuO nanoparticles. The transportation of nanoparticles is supposed to pass through the epidermis and cortex and finally to stele of the plant. It has been reported, perhaps for the first time, that the CuO nanoparticles were transported to the shoots and translocated back to the roots via phloem. It has also been shown that during the process of transportation of CuO nanoparticles to shoot via xylem and back to root via phloem, some of the Cu(II) in CuO is reduced to Cu(I). If this assumption is true, it may follow the reaction:

$$2\mathrm{CuO}\to {\mathrm{Cu}}_{2}O+O$$

$$O+O\to O_{2}$$

Since the authors have observed a blue colour after the addition [95] of Na_4_EDTA to CuO nanoparticles, it confirms the presence of Cu^2+^ rather than Cu^+1^ because Cu^+1^ having d^10^ configuration is colourless. This also confirms that the above hypothesis may not be true as it is not supplemented by experimental evidences. Root development of maize was inhibited by CuO nanoparticles followed by reduced biomass of the plant. The nanoparticles were distributed all over the plant parts which have adverse effect on them.

In an experiment with nanoparticles of different metal oxides on *Arabidopsis thaliana*, Lee et al. [161] have shown that all Al_2_O_3_, SiO_2_, Fe_3_O_4_ and ZnO are toxic. Seed germination, root elongation and leaf count were examined when seed or plants were exposed to concentrations of nanoparticles ranging from 400 to 4,000 mg L^-1^. The toxicity of metal oxide nanoparticles follows the order:

$$\mathrm{ZnO}>{\mathrm{Fe}}_{3}O_{4}>{\mathrm{SiO}}_{2}>{\mathrm{Al}}_{2}O_{3}$$

The solubility of ZnO nanoparticles is 33 times lower than the corresponding ZnCl_2_ in aqueous medium. It is surprising that while Zn^2+^ is a major constituent of over 30 enzymes in the human system, the ZnO-NP is toxic to *A. thaliana* even in very low concentration. Not all metal nanoparticles are useful to plants/animals, but some may be useful in some cases while others produce toxic effect. The seed germination was nearly inhibited but the leaves and roots did not grow at all in the presence of ZnO nanoparticles, while Fe_3_O_4_, SiO_2_ and Al_2_O_3_ nanoparticles had no marked influence at low concentration. It is stated by many workers that the toxicity of metal oxide nanoparticles may be caused by their dissolution and then the release of toxic metal ions [44,132,162]. However, it may happen only when known toxic metal nanoparticles such as Cd, Hg, Pd, As and Tl are taken. The innocuous types of metal oxide nanoparticles or metal nanoparticles in low concentration are not expected to produce adverse effect. It is also true that Zn being the most useful in mammalian system in low concentration may be toxic in higher concentration. A chemical in low concentration may act as medicine, but it may become poison when taken in bulk. Zn concentration up to 250 mg L^-1^ does not affect seed germination [161] which suggests that the phytotoxicity of metal oxide nanoparticles may be used to enhance or inhibit the plant growth (of certain type only). The influence of TiO_2_ and ZnO nanoparticles on seed germination, root length and number of roots of rice plant has been studied [163]. Although, there is no reduction in the percentage of germination of rice seed in both the cases, ZnO nanoparticles cause complete growth inhibition of the root. It cautions both agriculturist and environmentalist that dumping of waste disposal on the agricultural land may cause damage to the crops. As low as 400 mg L^-1^ ZnO nanoparticles inhibit root germination, and therefore, waste disposal at such places may be hazardous.

The toxic effect of CuO, NiO, TiO_2_, Fe_2_O_3_ and Co_3_O_4_ nanoparticles on germination, root elongation and growth of common edible plants such as lettuce, radish and cucumber has been done [164]. CuO and NiO nanoparticles at 12.9 and 27.9 mg L^-1^ concentration, respectively, are toxic to the above plants, while the other nanoparticles at such concentration are ineffective. The common trend of toxicity follows the order:

$$\mathrm{CuO}>\mathrm{NiO}>>{\mathrm{Fe}}_{2}O_{3}>{\mathrm{TiO}}_{2},{\mathrm{Co}}_{3}O_{4}$$

In some cases, TiO_2_ and SiO_2_ nanoparticles were found to enhance both the germination and growth of *Glycine max* seeds [129]. Carbon nanotubes (CNT) were found to enhance germination and root elongation of tomato seed [165] and produced two times more flowers and fruit [166]. Likewise, Al nanoparticles were found to be useful in augmenting the root of radish and rape seedlings [44]. Such effect depends on the concentration of nanoparticles and plant species under question. The CuO nanoparticle is not as much effective as free Cu^2+^ ions obtained from CuCl_2_. It is obvious that the quantity of Cu^2+^ ions released from CuO nanoparticles will be too small to be effective for germination of seeds.

The interaction of metal oxide nanoparticles with seed or plant tissue is poor comparative to free metal ions. The hypothesis that smaller nanoparticles can penetrate easily in plant cells and interact with biomolecules may not hold as the mobility of the particle may be the key factor. The small-sized nanoparticles will have higher degree of freedom for movement, and hence, they would be more efficiently absorbed by the plant.

Al_2_O_3_ nanoparticle has been shown to affect the plant growth and crop production. Phytotoxicity of Al_2_O_3_ nanoparticles was tested against five plant species [146]. When the same experiment was also run with Al_2_O_3_ loaded with phenanthrene (which is one of the hydrocarbons found in the atmosphere), it was found to be less toxic (root growth inhibition) than pure Al_2_O_3_. It suggests that Al_2_O_3_ nanoparticles may induce toxic effects on seedling root growth. However, submicron alumina particles loaded or unloaded with phenanthrene did not show any significant effect on seedling root growth. The decreased toxic effect of Al_2_O_3_ phenanthrene may be ascribed to size effect. Here, the nanoparticles accumulated and further accelerated due to phenanthrene which may have reduced the phytotoxicity of these particles. The FTIR spectrum of the particles showed bands in 850 to 1,050 cm^-1^ region which are assigned to vibrational modes of alumina [167].

$${\mathrm{Al}}_{2}O_{3}+3H_{2}O\to 2\mathrm{Al}{\left(\mathrm{OH}\right)}_{3}$$

**Figure 7:**
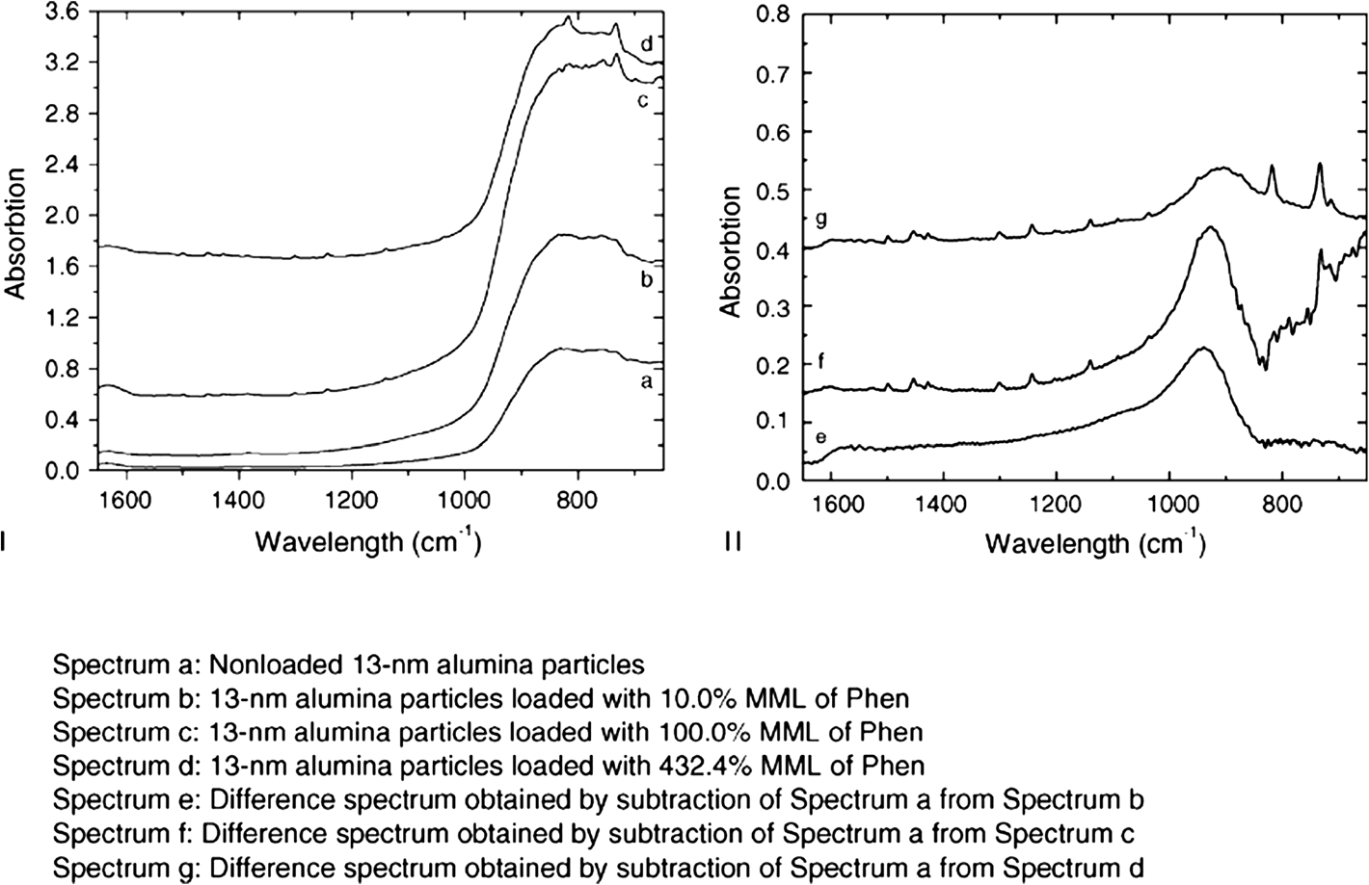
**FTIR spectra. I**: loaded particles (a); particles loaded with 10.0% (b), 100.0% (c) and 432.4% (d) monomolecular layer of phenanthrene. **II**: spectra obtained by subtraction of spectrum a from b, c and d, resulting in e, f and g, respectively. The band near 950 cm^-1^ is related to the surface characteristics of alumina nanoparticles [167]. The absorbance of phenanthrene can be distinguished in both spectra, f and g [146].

Since Al_2_O_3_ was taken in aqueous medium (either loaded or unloaded with phenanthrene), it may immediately react with water to give Al(OH)_3_. The FTIR spectrum will therefore, exhibit peak for Al-OH and not due to loss of hydroxyl group (Figure 7). The OH group may be lost if Al(OH)_3_ is heated in open according to

$$2\mathrm{Al}{\left(\mathrm{OH}\right)}_{3}\;\underset{△}{\to }{\mathrm{Al}}_{2}O_{3}+3H_{2}O$$

Pure Al_2_O_3_ may exhibit a peak due to Al-O. This assignment, on the basis of IR spectral data, may not be true. The authors [146] claim that dimethyl sulphoxide (DMSO) used in their experiment is a hydroxyl radical scavenger, and in aqueous medium, it removes the OH radical as shown below [168,169]:

$$\cdot \mathrm{OH}+{\left({\mathrm{CH}}_{3}\right)}_{2}\mathrm{SO}\to {\mathrm{CH}}_{3}\mathrm{SO}\left(\mathrm{OH}\right)+\cdot {\mathrm{CH}}_{3}$$

$$\cdot {\mathrm{CH}}_{3}+O_{2}\to {\mathrm{CH}}_{3}\mathrm{OO}\cdot$$

$$2{\mathrm{CH}}_{3}\mathrm{OO}\cdot \to \mathrm{HCHO}+{\mathrm{CH}}_{2}\mathrm{OH}+O_{2}$$

The last equation is wrong in the above reactions. It should produce CH_3_OH not CH_2_OH. Generally, free radicals combine with another species to give a molecule.

The effect of two fluorescent nanoparticles, fluorescein isothiocyanate (FITC)-silica nanoparticles and quantum dots (QD), on germination of rice seeds has been studied [170]. In addition, the uptake capacity of photostable CdSe QD and FITC-labelled silica nanoparticles (SNP) has also been studied. It was observed that germination in the presence of FITC-labelled SNP was enhanced while it was arrested with QD. Since the QD contain Cd as one of the known toxic metal ions, it may have reversibly acted on germination of rice seeds. However, transport of both fluorescent nanoparticles has been observed in rice seedlings. The FITC-SNP appears to be useful to plants and has shown good fluorescence in rice seedlings. It is therefore suggested that it may be used for bioimaging in plant tissues because of the photostability of SNP. Bioimaging can be done only with the help of fluorescent materials especially *in vivo*. Since very limited study has been done in this direction [171], the exact nature and mechanism of transport of nanoparticles is not well understood. It can equally be used in mammals, but the toxicity of such nanoparticles in biological system must be checked prior to its use. Conflicting reports have been received about the toxicity of QD [172,173] in mammals even though CdSe QD is known to arrest the root growth of rice seedlings.

**Figure 8:**
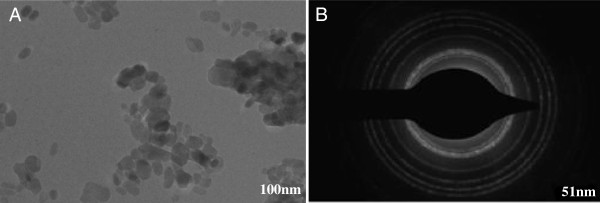
**TEM image (A) and SAED pattern (B) of nano-ZnO particles **[174]**.**

**Figure 9:**
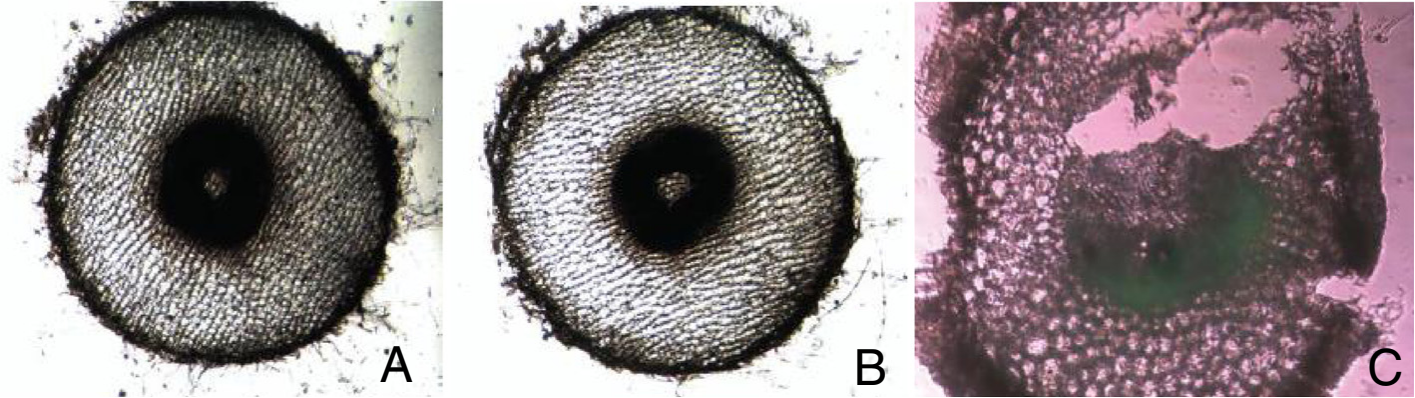
**Transverse section of *Cicer arietinum *seedling roots. (A)** Control, **(B)** at 1 ppm and **(C)** at 2,000 ppm of nano-ZnO treatment [174].

The useful application of metal or/and metal oxide nanoparticles is still a matter of controversy. In some cases, it has been found to be useful, while in many other instances, it appears to be phytotoxic [9-13]. The ZnO nanoparticles in this context have been used as growth promoter for *Cicer arietinum* and *Vigna radiata* seedlings [174]. They were monodispersed and their spherical shape was confirmed by SAED pattern (Figure 8). It was observed that in the case of *V. radiata* the root elongation occurs at 1-ppm level of ZnO nanoparticle while the shoot is almost unaffected when seedlings were exposed for 60 h. When the dose exceeds 1 to 20 ppm of ZnO, a sudden decrease in the shoot and root of *V. radiata* and *C. arietinum* seedlings occurs which is suggested to be the toxic level. From the analysis of ZnO nanoparticles in various parts of plant, it is found that the nanoparticles are absorbed and transported to other parts. Dispersion of epidermis, cortex and vascular cylinder was observed after higher concentration was administered (Figure 9). The adsorption and aggregation of ZnO nanoparticles in the root and damage to the architecture of the root were noted when a quantity above the optimum dose was given.

### Carbon nanomaterials and its beneficial and adverse effects

Carbon nanomaterials have received greater attention because of unique physical and chemical properties that enable the synthesis and manipulation to a degree not yet matched by inorganic nanostructures [175,176]. The effect of carbon nanomaterials of varying sizes and concentrations on different parts of a variety of plants has been studied [44,46,148,166,177-182]. Multi-walled carbon nanotubes (MWCNTs) enhanced alfalfa and wheat germination and root elongation, but the particle uptake and translocation was insignificant [183]. Increased root growth in response to carbon nanotubes was reported for onion, cucumber [177] and ryegrass [44]. MWCNTs have increased the growth of tobacco cells and tomato plants by affecting expression genes that are essential for cell division and plant development [166,184,185]. In addition to these, a number of other investigators have demonstrated toxicity of carbon nanomaterials to a range of plant species [46,186].

**Figure 10:**
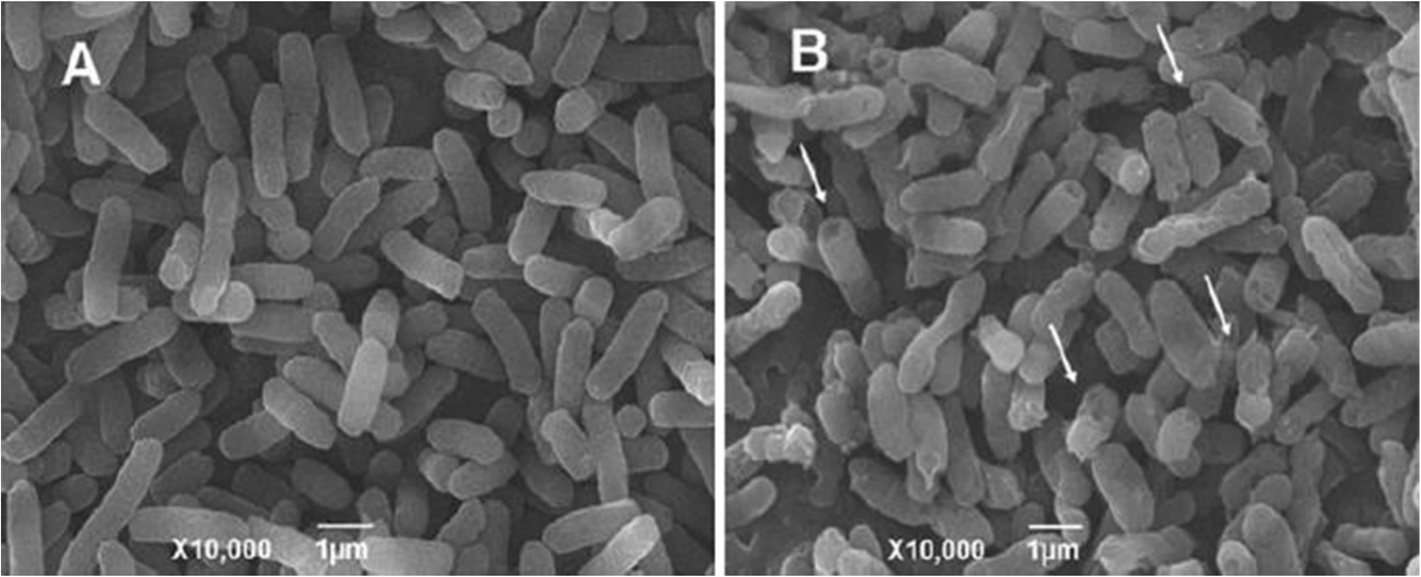
**Images of *E. coli *taken by SEM after exposure to nano-Ag. (A)** Control and **(B)** 1 μg mL^-1^ nano-Ag. Magnifications and plotting scales are marked out in each picture [193].

In an experiment, Mondal et al. [25] have shown that MWCNTs of approximately 30 nm diameter enhance the rate of germination and growth of *B. juncea*. Likewise, TiO_2_ nanoparticles have also been reported to enhance the rate of germination and strength of spinach seedlings [10]. Later, it was found in [165] that such nanoparticles increase the moisture contents of the seeds. The same is true with MWCNT which facilitates the reduction of water by adsorption and subsequent penetration into the seed coat and root of mustard plant. The oxidized CNT had better effect on the seed germination than the CNT alone, although the concentration of the oxidized CNT was much lower. Quite good results were obtained with oxidized MWCNT (2.3 × 10^-3^ mg mL^-1^), but when the concentration exceeds 46 × 10^-3^ mg mL^-1^, both MWCNT and oxidized MWCNT inhibit the germination of mustard seeds. It indicated that the rate of growth is concentration dependent. This technique may, therefore, be applied to increase the rate of germination of crop plants which reduces the time. It has been suggested that electrical conductivity of a solution increases when the plant tissues are immersed in it. This is correct up to a limit above which the conductance becomes constant because, as the concentration [187] of leached salts, amino acids, potassium, phosphate, sugar, carbohydrates, etc. increases, the freedom of movement of these molecules and ions decreases. Aquaporins are water channels that not only selectively allow water molecules to flow in and out of the tissue but also reject certain substances in order to maintain the equilibrium. It is concluded that pre-soaking of seeds with very low concentration of oxidized MWCNT have positive effect on seed germination. Exploitation of nanoparticles in different areas has become a fashionable trait even though their inadvertent use may create an imbalance in the ecosystem. For instance, Oberdörster [188] showed for the first time that the fullerenes, C_60_, cause lipid peroxidation in fish brain tissue, an example of adverse effect of nanoparticles in aquatic animals. Furthermore, fullerene (C_60_) is known for its multifunctional use such as imaging probe, antioxidant and drug carrier [189], but it has been shown to exhibit genotoxicity and cytotoxicity and also to induce ROS in rat/fish cell lines [190-192]. C_60_ can cause damage to *E. coli* but not to the extent of being used as a drug. On the other hand, an attempt to exploit it in other areas without knowing its properties may be hazardous. Wang et al. [193] studied the effect of gold, silver, iron and C_60_ nanoparticles on the growth of *E. coli*, *Bacillus subtilis* and *Agrobacterium tumefaciens*. It was observed that silver nanoparticle is most effective against all the above bacteria, while the other two nanoparticles have little or no influence on their growth. Perhaps, the silver nanoparticles easily penetrate the cell wall and interact with the pathogens inhibiting their further replication. The Au, Fe and C_60_ are regarded to be ineffective because they may be essential ingredients of these microbes. As little as 1 μg mL^-1^ silver nanoparticles are effective against the above bacterial strains. Approximately 5 μg mL^-1^ silver nanoparticles cause 100% mortality. It is clear from the SEM images that the cell wall of *E. coli* is damaged preventing further growth (Figure 10). In an experiment, Liu et al. [194] subjected human cell lines to silver nanoparticles of different sizes and demonstrated that smaller particles enter the cell more easily than the larger ones. Only penetration of nanoparticles into the cell wall is not the reason for their toxicity. It is concluded from a study that the toxicity of silver nanoparticles is due to their interaction with essential sulfhydryl group of the respiratory enzyme present in the bacterial cells [195].

The other assumption is that there is a formation of the free radical which induces membrane damage. The free radical is obviously very reactive, but the production of free radical requires homolytic fission of a species which may be linked to the protein. ROS may also be produced and may cause damage to the cell. The mechanism of action of silver nanoparticles with different cell lines is not yet clear, but it appears as if they adhere to the surface of bacterial cells leading to their mortality.

## Conclusions

The currently available information on nanomaterials suggests that it has great potential application in agri-food sectors, cosmetics (TiO_2_, ZnO, fullerene, Fe_2_O_3_ Cu, Ag, Au) catalyst (NiO, Pt, Pd) lubricants, fuel additives (CeO_2_, Pt, MoS_3_), paints and coatings (TiO_2_, SiO_2_, Ag, CdSe), agro-chemicals (SiO_2_), food packaging (Ag, TiO2. ZnO, TiN, nanoclay) nanomedicine and nanocarriers (Ag, Fe, magnetic materials). Nanotechnology offers a new range of benefits to food chain and human health by increasing the taste and flavour and reducing the amount of salt intake and fat thereby increasing the absorption and bioavailability of nutrients/supplements. Over 200 companies are conducting R&D into the application of nanotechnology in almost all areas. It has been estimated that about 150 applications of nanotechnology in food are at developmental stages and over 500 patents are in the pipeline. It is therefore anticipated that the use of nanotechnology will brighten the future prospect and enhance our knowledge with drastic reduction in the cost of nano-based food and medicines.

In conclusion, emphasis had been given to the phytosynthesis of nanoparticles from plant extract and their application in agriculture for substantial increase in biomass, fruit and crop yield especially in edible plants and vegetables such as cucumber, spinach, cabbage, radish, carrot, bitter melon and tomato. Many precious metals are also used as nanocatalyst to increase the production and decrease the cost. The drug delivery by nanomaterials is more important as the drug is quickly transported to the target cell without damaging the normal cells. Many nanomaterials are also essential plant nutrients and may therefore be absorbed to supplement deficiency in living system. Since with the minimum quantity of nanomaterial maximum yield is obtained, the disposal of nanomaterials will not create an environmental problem. This review is relevant in the present day scenario when there is an urgent need of enhanced food grain production to overcome its scarcity and to treat fatal diseases like cancer and AIDS.

## Competing interests

The authors declare that they have no competing interests.

## Authors' contributions

AH gathered the research data. AH and KSS analysed these data findings and wrote this review paper. Both authors read and approved the final manuscript.
